# Deep learning-based EEG emotion recognition: Current trends and future perspectives

**DOI:** 10.3389/fpsyg.2023.1126994

**Published:** 2023-02-27

**Authors:** Xiaohu Wang, Yongmei Ren, Ze Luo, Wei He, Jun Hong, Yinzhen Huang

**Affiliations:** ^1^School of Intelligent Manufacturing and Mechanical Engineering, Hunan Institute of Technology, Hengyang, China; ^2^School of Electrical and Information Engineering, Hunan Institute of Technology, Hengyang, China; ^3^School of Computer and Information Engineering, Hunan Institute of Technology, Hengyang, China

**Keywords:** human–computer interaction, electroencephalogram, emotion recognition, deep learning, survey

## Abstract

Automatic electroencephalogram (EEG) emotion recognition is a challenging component of human–computer interaction (HCI). Inspired by the powerful feature learning ability of recently-emerged deep learning techniques, various advanced deep learning models have been employed increasingly to learn high-level feature representations for EEG emotion recognition. This paper aims to provide an up-to-date and comprehensive survey of EEG emotion recognition, especially for various deep learning techniques in this area. We provide the preliminaries and basic knowledge in the literature. We review EEG emotion recognition benchmark data sets briefly. We review deep learning techniques in details, including deep belief networks, convolutional neural networks, and recurrent neural networks. We describe the state-of-the-art applications of deep learning techniques for EEG emotion recognition in detail. We analyze the challenges and opportunities in this field and point out its future directions.

## 1. Introduction

Emotion recognition (or detection) is a major scientific problem in affective computing, which mainly solves the problem of computer systems accurately processing, recognizing, and understanding the emotional information expressed by human beings. Affective computing requires interdisciplinary knowledge, including psychology, biology, and computer science. As emotion plays a key role in the field of human–computer interaction (HCI) and artificial intelligence, it has recently received extensive attention in the field of engineering research. Research of emotion recognition technology can further promote the development of various disciplines, including computer science, psychology, neuroscience, human factors engineering, medicine, and criminal investigation.

As a complex psychological state, emotion is related to physical behavior and physiological activities (Cannon, 1927). Researchers have conducted numerous studies to enable computers to correctly distinguish and understand human emotions. These studies aim to enable computers to generate various emotional features similar to human beings, so as to achieve the purpose of natural, sincere, and vivid interaction with human beings. Some of these methods mainly use non physiological signals, such as speech (Zhang et al., 2017; Khalil et al., 2019; Zhang S. et al., 2021), facial expression (Alreshidi and Ullah, 2020), and body posture (Piana et al., 2016). However, their accuracy depends on people’s age and cultural characteristics, which are subjective, so accurately judging the true feelings of others is difficult. Other methods use physiological activities (or physiological clues), such as heart rate (Quintana et al., 2012), skin impedance (Miranda et al., 2018), respiration (Valderas et al., 2019) or brain signals, functional magnetic resonance imaging (Chen et al., 2019a), magnetoencephalography (Kajal et al., 2020), and electroencephalography, to identify emotional states. Some studies have shown that physiological activities and emotional expression are correlated, although the sequence of the two processes is still debated (Cannon, 1927). Therefore, the method based on calculating physiological signals is considered an effective supplement to the recognition method based on nonphysiological signals. The subject cannot control the automatically generated electroencephalogram (EEG) signal. For those who cannot speak clearly and express their feelings through natural speech or have physical disabilities and cannot express their feelings through facial expressions or body postures, emotion recognition of voice, expression, and posture becomes impossible. Therefore, EEG is an appropriate means to extract human emotions, and studying emotional cognitive mechanisms and recognizing emotional states by directly using brain activity information, such as EEG, are particularly important.

From the perspective of application prospects, EEG-based emotion recognition technology has penetrated into various fields, including medical, education, entertainment, shopping, military, social, and safe driving (Suhaimi et al., 2020). In the medical field, timely acquisition of patients’ EEG signals and rapid analysis of their emotional state can help doctors and nurses to accurately understand the patients’ psychological state and then make reasonable medical decisions, which has an important effect on the rehabilitation of some people with mental disorders, such as autism (Mehdizadehfar et al., 2020; Mayor-Torres et al., 2021; Ji et al., 2022), depression (Cai et al., 2020; Chen X. et al., 2021), Alzheimer’s disease (Güntekin et al., 2019; Seo et al., 2020), and physical disabilities (Chakladar and Chakraborty, 2018). In terms of education, the emotion recognition technology based on EEG signals can enable teaching staff to adjust teaching methods and teaching attitudes in a timely manner in accordance with the emotional performance of different trainees in class, such as increasing or reducing the workload (Menezes et al., 2017). In terms of entertainment, such as computer games, researchers try to detect the emotional state of players to adapt to the difficulty, punishment, and encouragement of the game (Stavroulia et al., 2019). In the military aspect, the emotional status of noncommissioned officers and soldiers can be captured timely and accurately through EEG signals, so that the strategic layout can be adjusted in time to improve the winning rate of war (Guo et al., 2018). In terms of social networks, we can enhance barrier-free communication in the HCI system, increase the mutual understanding and interaction in the human–machine–human interaction channel, and avoid some unnecessary misunderstandings and frictions through the acquisition of emotional information (Wu et al., 2017). In terms of safe driving, timely detection of EEG emotional conditions can enable a vehicle to perform intelligent locking during startup to block driving or actively open the automatic driving mode to intervene in the vehicle’s motion trajectory until parking at a safe position, thereby greatly reducing the occurrence of accidents (Fan et al., 2017).

**Figure 1:**
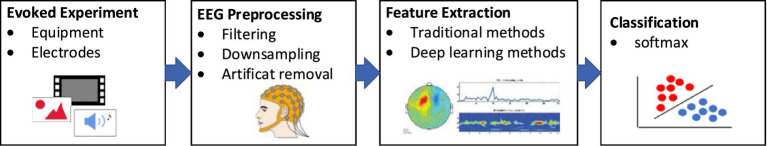
Flowchart of emotion recognition using EEG signals.

Recently, automatic recognition of emotional information from EEG has become a challenging problem, and has attracted extensive attention in the fields of artificial intelligence and computer vision. The flow of emotion recognition research is shown in Figure 1. Essentially, human emotion recognition using EEG signals belongs to one type of pattern recognition research.

In the early EEG-based automated emotion recognition literature, a variety of machine learning-based studies, such as support vector machine (SVM; Lin et al., 2009; Nie et al., 2011; Jie et al., 2014; Candra et al., 2015), k-nearest neighbor (KNN; Murugappan et al., 2010; Murugappan, 2011; Kaundanya et al., 2015), linear regression (Bos, 2006; Liu et al., 2011), support vector regression (Chang et al., 2010; Soleymani et al., 2014), random forest (Lehmann et al., 2007; Donos et al., 2015; Lee et al., 2015), and decision tree (Kuncheva et al., 2011; Chen et al., 2015), have been developed.

Although the abovementioned hand-crafted EEG signal features associated with machine learning approaches can produce good domain-invariant features for EEG emotion recognition, they are still low-level and not highly discriminative. Thus, obtaining high-level domain-invariant feature representations for EEG emotion recognition is desirable.

**Figure 2:**
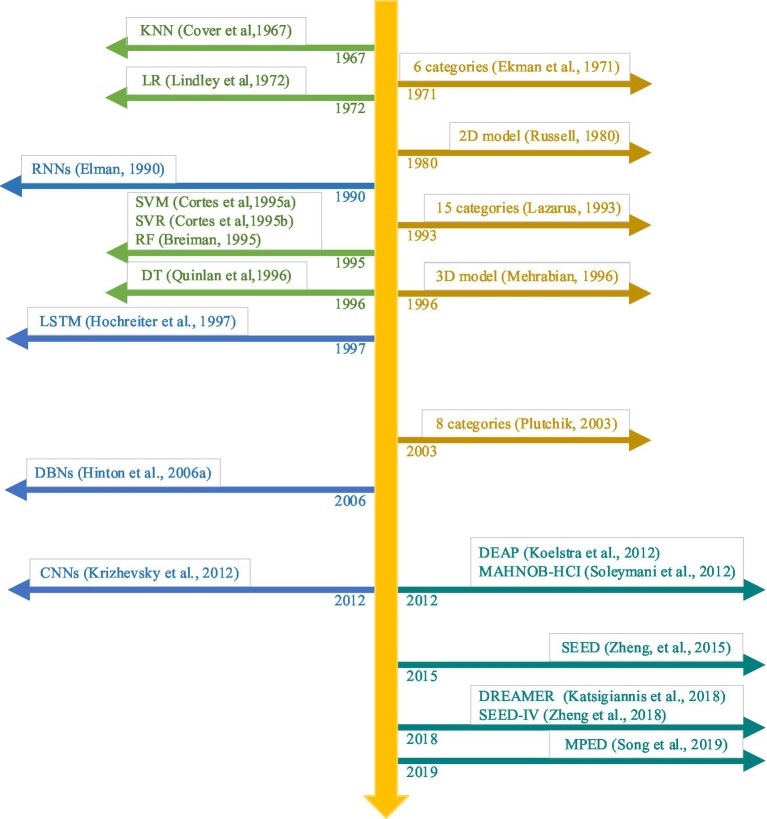
The evolution of EEG emotion recognition with deep learning algorithms, emotion categories and databases.

The recently-emerged deep learning methods may present a possible solution to achieve high-level domain-invariant feature representations and high-precision classification results of EEG emotion recognition. The representative deep leaning techniques contain recurrent neural networks (RNNs; Elman, 1990), long short-term memory (LSTM; Hochreiter and Schmidhuber, 1997; Zhang et al., 2019), deep belief networks (DBNs; Hinton et al., 2006), and convolutional neural networks (CNNs; Krizhevsky et al., 2012). To date, deep learning techniques have shown outstanding performance on object detection and classification (Wu et al., 2020), natural language processing (Otter et al., 2020), speech signal processing (Purwins et al., 2019), and multimodal emotion recognition (Zhou et al., 2021) due to its strong feature learning ability. Figure 2 shows the evolution of EEG emotion recognition with deep learning algorithms, emotion categories and databases.

Inspired by the lack of summarizing the recent advances in various deep learning techniques for EEG-based emotion recognition, this paper aims to present an up-to-date and comprehensive survey of EEG emotion recognition, especially for various deep learning techniques in this area. This paper highlights the different challenges and opportunities on EEG emotion recognition tasks and points out its future trends. In this survey, we have searched the published literature between January 2012, and December 2022 through Scholar. google, ScienceDirect, IEEEXplore, ACM, Springer, PubMed, and Web of Science, on the basis of the following keywords: “EEG emotion recognition,” “emotion computing,” “deep learning,” “RNNs,” “LSTM,” “DBNs,” and “CNNs.” There is no any language restriction for the searching process.

In this work, our contributions can be summarized as follows:

1. We provide an up-to-date literature survey on EEG emotion recognition from a perspective of deep learning. To the best of our knowledge, this is the first attempt to present a comprehensive review covering EEG emotion recognition and deep learning-based feature extraction algorithms in this field.
2. We analyze and discuss the challenges and opportunities faced to EEG emotion recognition and point out future directions in this field.

The organization of this paper is as follows. We first present the preliminaries and basic knowledge of EEG emotion recognition. We review benchmark datasets and deep learning techniques in detail. We show the recent advances of the applications of deep learning techniques for EEG emotion recognition. We give a summary of open challenge and future directions. We provide the concluding remarks.

## 2. Preliminaries and basic knowledge

### 2.1. Definition of affective computing

Professor Picard (1997) of the MIT and his team clearly defined affective computing, that is, the calculation of factors triggered by emotion, related to emotion, or able to affect and determine emotional change. In accordance with the research results in the field of emotion, emotion is a mechanism gradually formed in the process of human adaptation to social environment. When different individuals face the same environmental stimulus, they may have the same or similar emotional changes or they may have different emotional changes due to the difference in individual living environment. This psychological mechanism can play a role in seeking advantages and avoiding disadvantages. Although computers have strong logic computing ability, human beings cannot communicate more deeply when interacting with computers due to the lack of psychological mechanisms similar to human beings. Emotion theory is an effective means to solve this problem. Therefore, an effective method to realize computer intelligence is to combine logical computing with emotional computing, which is a research topic that many researchers focus on at present (Zhang S. et al., 2018).

### 2.2. Classification of emotional models

Many researchers cannot reach a unified emotional classification standard when conducting emotional computing research due to the high complexity and abstractness of emotion. At present, researchers usually divide emotion models into discrete model and dimensional space model.

In the discrete model, each emotion is distributed discretely, and these discrete emotions combine to form the human emotional world. In the discrete model, designers have different definitions of emotions, and they are divided into different emotional categories. American psychologist Ekman and Friesen (1971) divided human emotions into six basic emotions, namely, anger, disgust, fear, happiness, sadness, and surprise, by analyzing human facial expressions. Lazarus (Lazarus, 1993), one of the modern representatives of American stress theory, divided emotions into 15 categories, such as anger, anxiety and happiness, and each emotional state has a corresponding core related theme. Psychologist Plutchik (2003) divided emotions into eight basic categories: anger, fear, sadness, disgust, expectation, surprise, approval, and happiness. These discrete emotion classification methods are relatively simple and easy to understand, and have been widely used in many emotion recognition studies.

**Figure 3:**
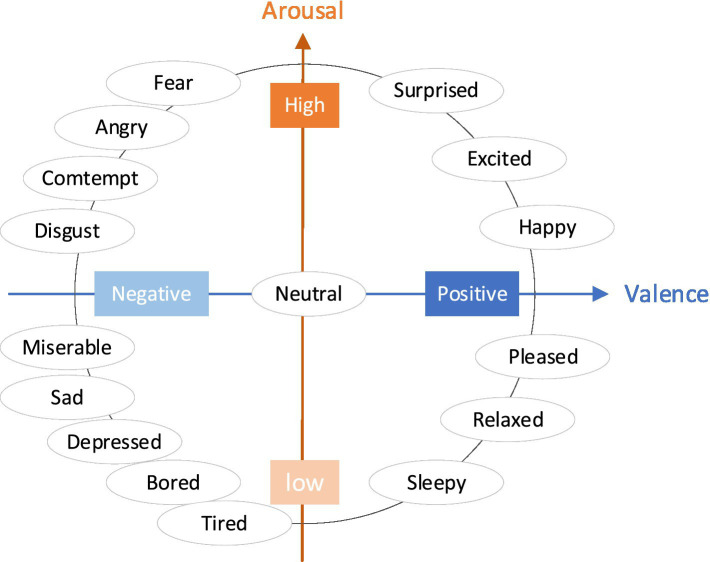
Two-dimensional model for valence–arousal.

The dimensional space model of emotion can be divided into 2D and 3D. The 2D expression model of emotion was first proposed by psychologist Russell (1980). It uses 2D coordinate axis to describe the polarity and intensity of emotion. Polar axis is used to describe the positive and negative types of emotion, and intensity coordinate axis refers to the intensity of emotion. The 2D emotion model is consistent with people’s cognition of emotion. Currently, the VA model that divides human emotions into two dimensions is widely used, which are the valency dimension and arousal dimension, as shown in Figure 3.

**Figure 4:**
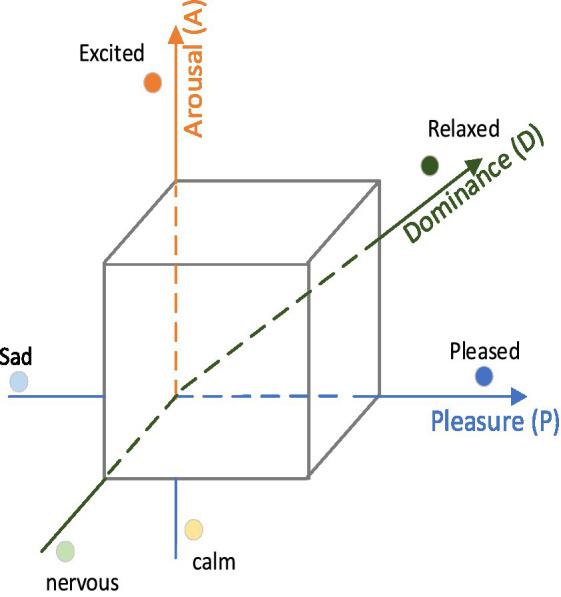
PAD 3D emotional model.

Considering that the 2D space representation of emotions cannot effectively distinguish some basic emotions, such as fear and anger, Mehrabian (1996) proposed a 3D space representation of emotions, and its three dimensions are pleasure, activation, and dominance, as shown in Figure 4. Centered on the origin, pleasure (P) represents the difference between positive and negative emotions; arousal (A) indicates the activation degree of human emotions; dominance (D) indicates the degree of human control over current things. At the same time, the coordinate values of the three dimensions can be used to describe specific human emotions.

### 2.3. Deep learning techniques

#### 2.3.1. DBNs

DBNs proposed by Hinton et al. (2006) are a generative model aim to train the weights among its neurons and make the entire neural network generate training data in accordance with the maximum probability.

At present, DBN has been applied to many areas of life, such as voice, graphics, and other visual data classification tasks, and achieved good recognition results. Tong et al. used a DBN model to classify hyperspectral remote sensing images, improved the training process of DBN, and used the hyperspectral remote sensing image dataset Salinas to verify the proposed method (Tong et al., 2017). Compared with traditional model classification methods, the classification accuracy of DBN model can reach more than 90%. In the text classification event, Payton L et al. proposed an ME learning algorithm for DBN (Payton et al., 2016). This algorithm is specially designed to deal with limited training data. Compared with the maximum likelihood learning method, the method of maximizing the entropy of parameters in DBN has more effective generalization ability, less data distribution deviation, and robustness to over fitting. It achieves good classification effect on Newsgroup, WebKB, and other datasets. The DBN model also achieves good classification results in speech classification events. Wen et al. tried to recognize human emotions from speech signals (Wen et al., 2017) using a random DBN (RDBN) integrating method. The experimental results on the benchmark speech emotion database show that the accuracy of RDBN is higher than that of KNN and other speech emotion recognition methods. Kamada S and Ichimura T extended the learning algorithm of adaptive RBM and DBN to time series analysis by using the idea of short-term memory and used the adaptive structure learning method to search the optimal network structure of DBN in the training process. This method was applied to MovingMNIST, a benchmark dataset for video recognition, and its prediction accuracy exceeded 90% (Kamada and Ichimura, 2019).

#### 2.3.2. CNNs

The concept of neural networks originated from the neural mathematical model first proposed in 1943 (Mcculloch and Pitts, 1943). However, the artificial neural networks confined to the shallow network architecture fell into a low tide in the late 1960s due to the constraints of early computing power, data and other practical conditions. The real rise of neural network method began with AlexNet (Krizhevsky et al., 2012) proposed by Hinton et al. (2012). This CNN model won the Image Net Large Scale Visual Recognition Challenge (Deng et al., 2009) with a huge advantage of 10.9%. Therefore, deep neural networks (DNNs) have gradually attracted extensive attention from the industry and academia. In a broad sense, the DNNs can be divided into feedforward neural networks (Rumelhart et al., 1986; Lecun and Bottou, 1998) and RNNs (Williams and Zipser, 1989; Yi, 2010; Yi, 2013) in accordance with the difference in connection modes between neurons. In accordance with the differences in use scenarios and HCI methods, the DNNs can be divided into single input networks and multi-input networks, which are continuously extended to a variety of HCI scenarios and have achieved breakthrough results (Wang et al., 2018), allowing AI products to be actually implemented in practical applications.

#### 2.3.3. RNNs

Compared with CNNs, RNNs are better in processing data with sequence characteristics and can obtain time-related information in data (Lecun et al., 2015). Researchers have widely used RNNs in natural language processing, including machine translation, text classification, and named entity recognition (Schuster and Paliwal, 1997). RNNs have achieved outstanding performance in audio-related fields and made great breakthroughs. They have been widely used in speech recognition, speech synthesis, and other fields. Considering that RNN only considers the preorder information and ignores the postorder information, a bidirectional RNN (BRNN) was proposed (Schuster and Paliwal, 1997). To solve the problems of gradient disappearance and gradient explosion in the training process of the long sequence of RNN, researchers improved the structure of RNN and built a LSTM (Hochreiter and Schmidhuber, 1997). LSTM has modified the internal structure of RNN in the current time step, making the hidden layer architecture more complex, which can have a better effect in longer sequences and is a more widely used RNN in the general sense.

The bidirectional LSTM (BILSTM) can be obtained by combining LSTM and BRNN (Graves and Schmidhuber, 2005). It replaces the original RNN neuron structure in BRNN with the neuron structure of LSTM and combines the forward LSTM and backward LSTM to form a network. BILSTM retains the advantages of BRNN and LSTM at the same time. It can retain the context information of the current time node and record the relationship between the front and back features. Therefore, BILSTM improves the generalization ability of the network model and the ability to handle long sequences, and avoids the problem of gradient explosion and gradient disappearance according to the difference of connection modes between neurons.

In recent years, gated recurrent unit (GRU) networks (Cho et al., 2014) were proposed. They discard the three LSTM gated (force gate, input gate, and output gate) networks, selects reset gate and update gate, and combines the current state of neurons and the hidden layer state, which are uniformly expressed as *h_t_*. Compared with LSTM, the GRU model is simpler, with fewer parameters and easier convergence.

## 3. Benchmark datasets

The proposed EEG emotion recognition algorithms should be verified on EEG data with emotion ratings or labels. However, some researchers are limited by conditions and cannot build a special experimental environment. Most researchers are interested in verifying their algorithms and comparing with relevant studies on the recognized benchmark datasets. Hence, a variety of open-source EEG emotion databases have been developed for EEG emotion recognition. Table 1 presents a brief summary of existing speech emotion databases. In this section, we describe briefly these existing EEG emotion databases as follows.

**Table 1:** Description of public datasets.

Name	Participants	Documented Signals	Stimulus	Task models/ Emotions
DEAP	32	EEG, EMG, EOG, GSR, Temperature, and Face Video	40 Video clips	VAD model
MAHNOB-HCI	27	EEG, ECG, GSR, ERG, Respiration Amplitude, Skin Temperature, Face Video, Audio Signals, and Eye Gaze	20 Video clips and Pictures	VAD model
SEED	15	EEG, Face Video, and Eye tracking	15 Video clips	Positive, Neutral, and Negative
DREAMER	23	EEG, ECG	18 Video clips	VAD model
SEED-IV	15	EEG, and EM	168 Video clips	Happiness, Sadness, Fear and Neutrality
MPED	23	EEG、ECG、RSP、and GSR	28 Video clips	Joy, Funny, Anger, Fear, Disgust, Sadness, and Neutrality

### 3.1. Database for emotion analysis using physical signals

**Figure 5:**
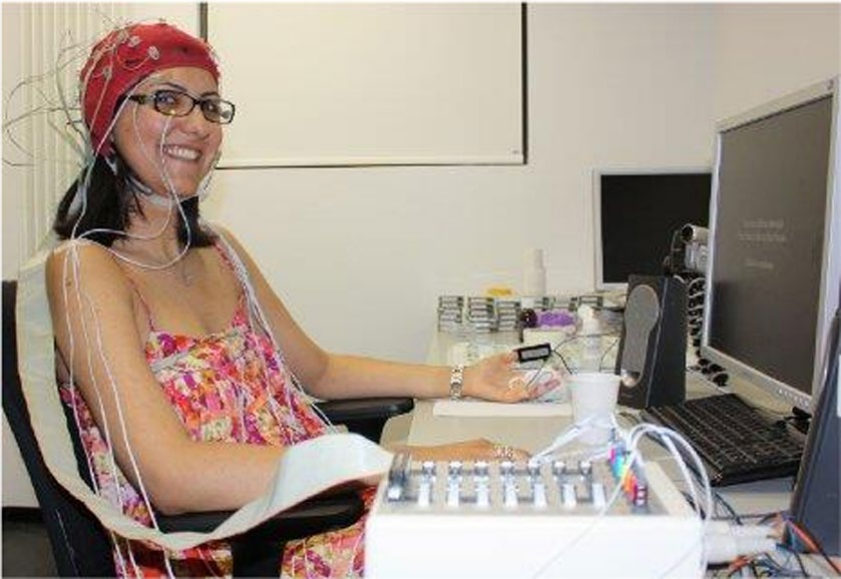
Collection scene for the Deap database (Koelstra et al., 2012). Reproduced with permission from IEEE. Licence ID: 1319273-1.

DEAP is a large multimodal physiological and emotional database jointly collected and processed by Koelstra and other research institutions of four famous universities (Queen Mary University in London, Twente University in the Netherlands, Geneva University in Switzerland, and Swiss Federal Institute of Technology; Koelstra et al., 2012). The collection scene for the DEAP database is shown in Figure 5, and it is an open-source data set for analyzing human emotional states. The DEAP database collected 32 participants for the experiment, where 16 of them were male and 16 were female. In the experiment, the EEG and peripheral physiological signals of the participants were collected, and the frontal facial expression videos of the first 22 participants were recorded. The participants read the instructions of the experiment process and wore the detection equipment before starting the data acquisition experiment. Each participant watched 40 music video clips with a duration of 1 min in the experiment. The subjective emotional experience in induction experiments was self-evaluated and rated on assessment scales that cover four emotional dimensions, namely, arousal, valence, dominance, and like. During self-assessment, the participants saw the content displayed on the screen and clicked to select the option that matched their situation at that time. The EEG and peripheral physiological signals were recorded by using a Biosemi ActiveTwo system. The EEG information was collected by using electrode caps with 32 AgCl electrodes. The EEG sampling rate was 512 Hz. The data set recorded 40 channels in total, the first 32 channels were EEG signal channels, and the last 8 channels were peripheral signal channels.

### 3.2. Multimodal database for affect recognition and implicit tagging

**Figure 6:**
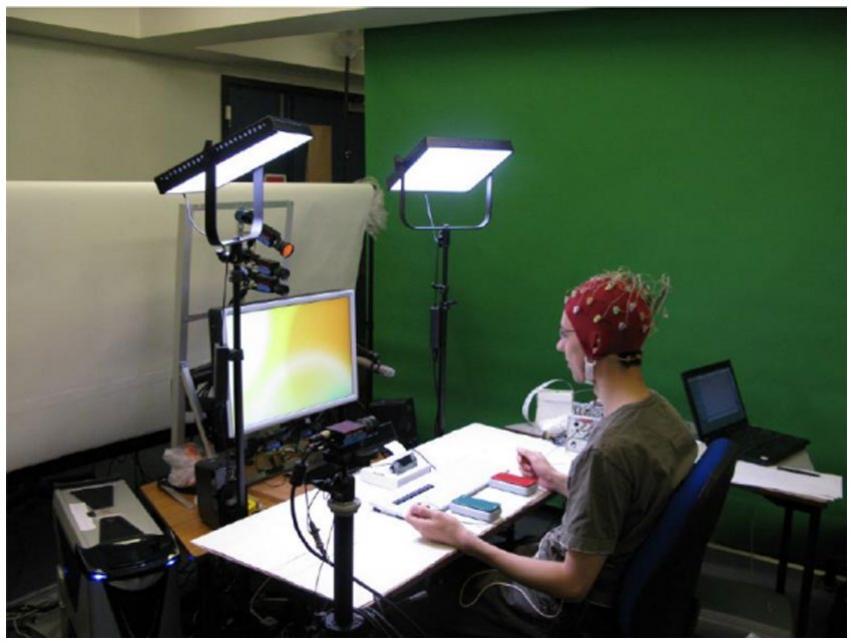
Collection scene for the MAHNOB-HCI database (Soleymani et al., 2014). Reproduced with permission from IEEE. Licence Number: 5493400200365.

MAHNOB-HCI is a multimodal physiological emotion database collected by Soleymani et al. (2014) through a reasonable and normal experimental paradigm. The MAHNOB-HCI dataset collected EEG signals and peripheral physiological signals from 30 volunteers with different cultural and educational backgrounds using emotional stimulation videos. Among the 30 young healthy adult participants, 17 were women and 13 were men, and the age ranged from 19 to 40. Thirty participants watched 20 different emotional video clips selected from movies and video websites. These video clips can stimulate the subjects to have five emotions: disgust, amusement, fear, sadness, and joy. The duration of watching videos was 35 to 117 s. The participants evaluated the arousal and potency dimensions rated on assessment scales after watching each video clip. In the data collection experiment, six cameras were used to record the facial expressions of the subjects at a frame rate of 60 frames per second. The collection scene for the MAHNOB-HCI database is shown in Figure 6.

### 3.3. SJTU emotion EEG dataset

**Figure 7:**
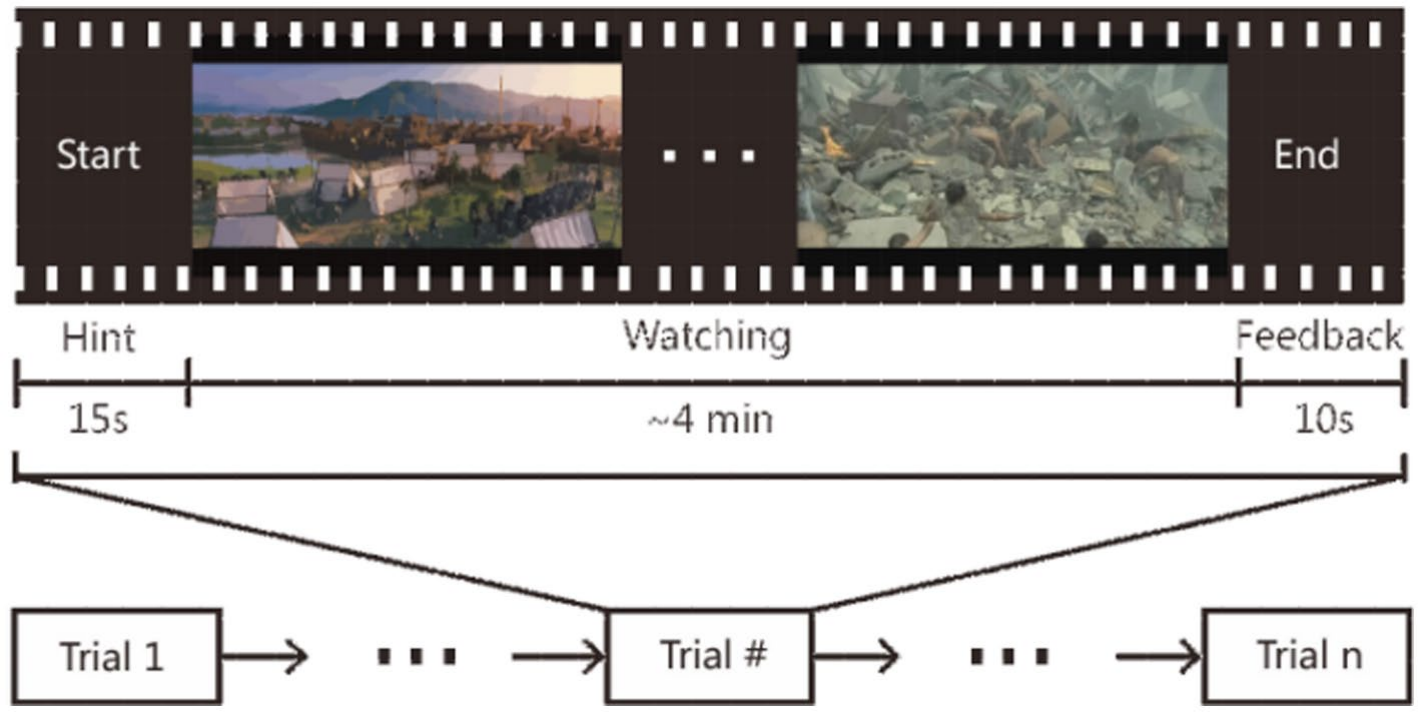
Protocol used in the emotion experiment (Zheng and Lu, 2015). Reproduced with permission from IEEE. Licence Number: 5493480479146.

The SEED is an EEG emotion dataset released by the BCMI Research Center in Shanghai Jiaotong University (SJTU; Zheng and Lu, 2015), and the protocol used in the emotion experiment is shown in Figure 7. The SEED dataset selected 15 people (7 men and 8 women) as the subjects of the experiment and collected data of 62 EEG electrode channels from the participants. In the experiment, 15 clips of Chinese movies were selected for the subjects to watch. These videos contained three types of emotions: positive, neutral, and negative. Each genre had five clips, and each clip was about 4 min. Clips containing different emotions appeared alternately. In the experiment, the subjects had a 5-s prompt before watching each video. The subjects conducted a 45 s self-assessment, followed by a 15 s rest. During the experiment, the subjects were asked to complete three experiments repeatedly in a week or even longer. Each subject watched the same 15 clips of video and recorded their self-evaluation emotions.

### 3.4. Dreamer

The Dreamer database (Katsigiannis and Ramzan, 2018) is a multimodal physiological emotion database released by the University of Western Scotland in 2018. It contains 18 audio-visual clips during the emotion induction experiments and collects the EEG and electrocardiogram (ECG) signals simultaneously. The video duration is between 65 and 393 s, with an average duration of 199 s. Twenty-three subjects with an average age of 26.6 years were invited to participate in the experiment. The subjects were asked to conduct a self-assessment between 1 and 5 points in the emotional dimensions of valence, arousal, and dominance after each emotional induction experiment.

### 3.5. Seed-iv

**Figure 8:**
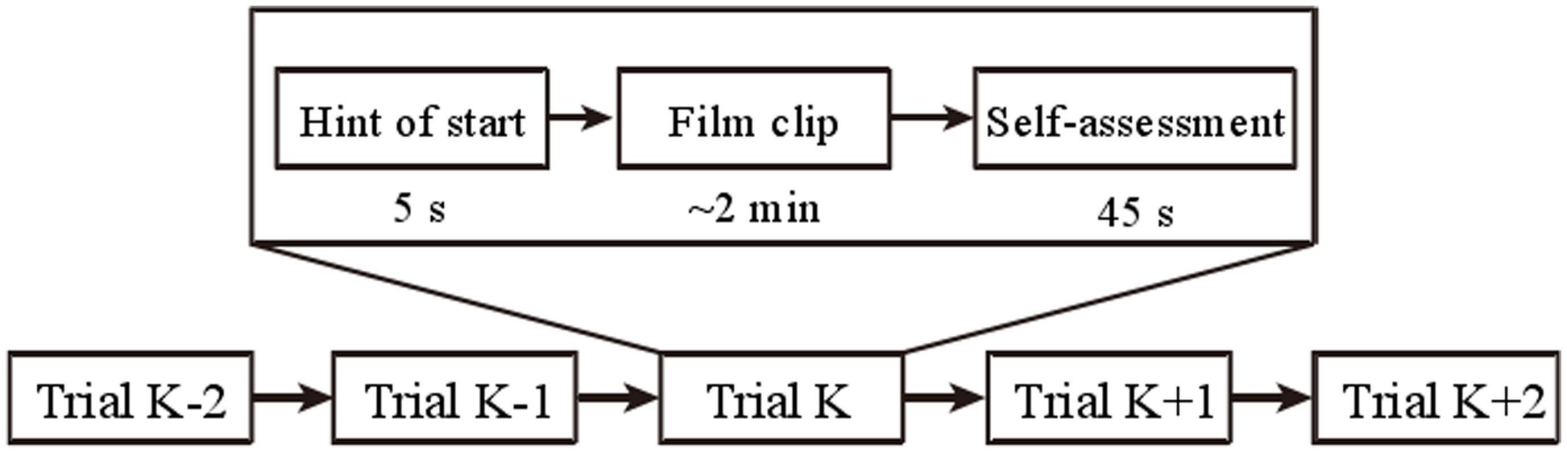
Protocol of SEED-IV for four emotions (Zheng et al., 2018). Reproduced with permission from IEEE. Licence Number: 5493600273300.

SEED-IV is another version of the SEED dataset released by SJTU (Zheng et al., 2018), which has been widely used in recent related work. The protocol of SEED-IV for four emotions is shown in Figure 8. Forty-four participants (22 women, all college students) were recruited to self-evaluate their emotions during the induction experiment, and 168 film clips were selected as the material library of four emotions (happiness, sadness, fear, and neutrality). It follows the experimental paradigm adopted in SEED, 62-channel EEG of 15 selected subjects were recorded in the three tests. They chose 72 film clips with four different emotional labels (neutral, sad, fear, and happy). Each subject watched six film clips in each session, resulting in 24 trials in total.

### 3.6. Multi-modal physiological emotion database

The MPED contains four physiological signals of 23 subjects (10 men and 13 women) and records seven types of discrete emotion (joy, funny, anger, fear, disgust, sadness, and neutrality) when they watch 28 video clips (Song et al., 2019). The experiments are divided into two sessions with an interval of at least 24 h. Twenty-one EEG data are used as training data, and the remaining 7 EEG data are used as test data.

## 4. Review of EEG emotion recognition techniques

### 4.1. Shallow machine learning methods for EEG emotion recognition

The emotion recognition method of EEG signals based on machine learning is usually divided into two steps: manual feature extraction and classifier selection (Li et al., 2019). The feature extraction methods mainly include time domain analysis, frequency domain analysis, time frequency domain analysis, multivariate statistical analysis, and nonlinear dynamic analysis (Donos et al., 2018; Rasheed et al., 2020). Principal component analysis, linear discriminant analysis (LDA; Alotaiby et al., 2017), and independent component analysis (Ur et al., 2013; Acharya et al., 2018a; Maimaiti et al., 2021) are widely used unsupervised time-domain methods to summarize EEG data. Frequency domain features include spectral center, coefficient of variation, power spectral density, signal energy, spectral moment, and spectral skewness, which can provide key information about data changes (Yuan et al., 2018; Acharya et al., 2018a). The abovementioned time-domain or frequency-domain methods have limitations and cannot provide accurate frequency or time information at a specific time point. Wavelet transformation (WT) is usually used to decompose EEG signals into their frequency components to express the relationship between signal information and time. Time frequency signal processing algorithms, such as discrete wavelet transform analysis and continuous wavelet transform, are a necessary means to solve different EEG behavior, which can be described in the time and frequency domains (Martis et al., 2012; UR et al., 2013). Statistical parameters, such as mean, variance, skewness, and kurtosis, have been widely used to extract feature information from EEG signals. Variance represents the distribution of data, skewness represents the symmetry information of data, and kurtosis provides the peak information in data (Acharya et al., 2018a).

In classifier selection, previous work mainly used shallow machine learning methods, such as LDA, SVM, and KNN, to train emotion recognition models based on manual features. Although the method of “manual features+shallow classifier” has made some progress in previous emotion recognition systems, the design of manual features requires considerable professional knowledge, and the extraction of some features (such as linear features) is time consuming.

### 4.2. Deep learning for EEG emotion recognition

**Figure 9:**
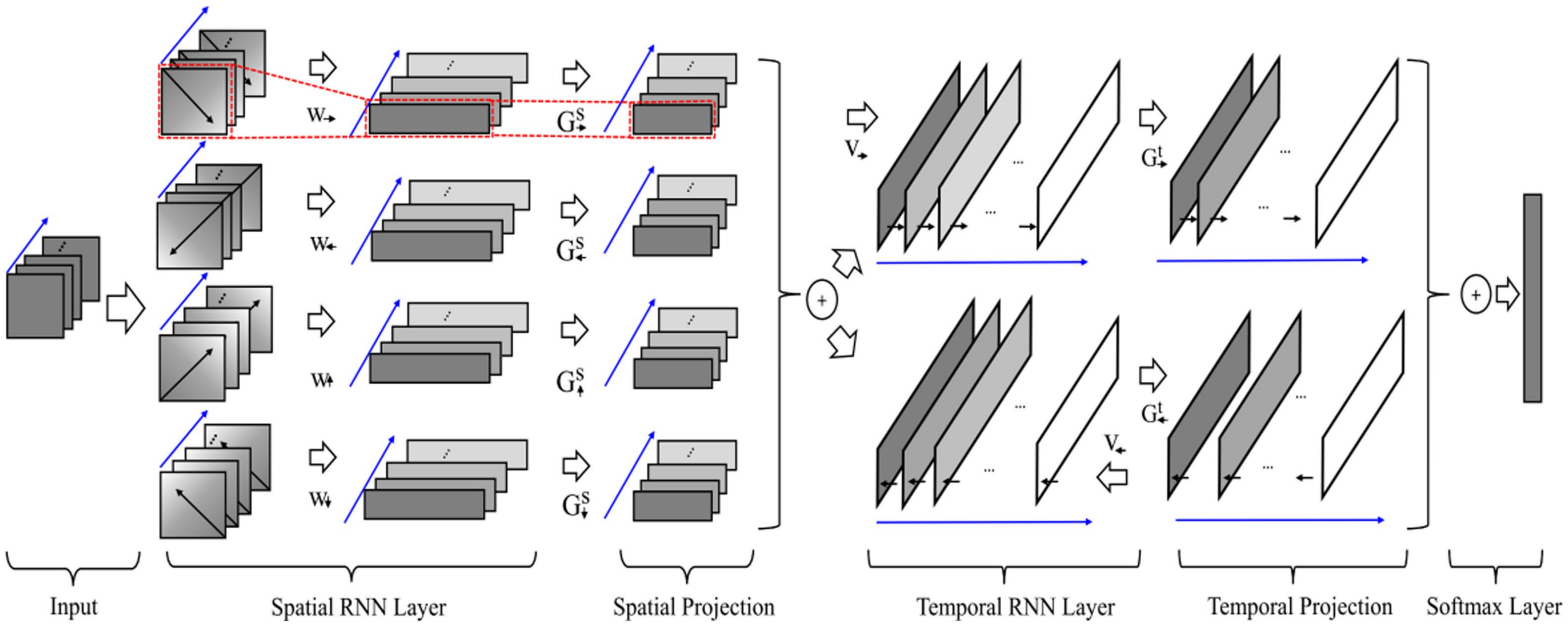
The used STRNN framework for EEG emotion recognition (Zhang T. et al., 2018). Reproduced with permission from IEEE. Licence Number: 5493591292973.

Traditional machine learning techniques extract EEG features manually, which not only have high redundancy in the extracted features, but also have poor universality. Therefore, manual feature extraction techniques can not achieve the ideal results in EEG emotion recognition. Obviously, with the increasing progress of deep learning technology (Chen B. et al., 2021), EEG emotion recognition research ground on various neural networks has gradually become a research hotspot. Different from shallow classifier, deep learning has the advantages of strong learning ability and good portability, which can automatically learn good feature representations instead of manually design. Recently, various deep learning models, such as DNN, CNN, LSTM, and RNN models, were tested on public datasets. Compared with CNN, RNN is more suitable for processing sequence-related tasks. LSTM has been proven to be capable of capturing time information in the field of emotion recognition (Bashivan et al., 2015; Ma et al., 2019). As a type of sequence data, most studies on EEG are based on RNN and LSTM models. Li et al. (2017) designed a hybrid deep learning model by combining CNN and RNN to mine inter-channel correlation. The results demonstrated the effectiveness of the proposed methods, with respect to the emotional dimensions of Valence and Arousal. Zhang T. et al. (2018) proposed a spatial–temporal recurrent neural network (STRNN) for emotion recognition, which integrate the feature learning from both spatial and temporal information of signal sources into a unified spatial–temporal dependency model, as shown in Figure 9. Experimental results on the benchmark emotion datasets of EEG and facial expression show that the proposed method is significantly better than those state-of-the-art methods. Nath et al. (2020) compared the emotion recognition effects of LSTM with KNN, SVM, DT, and RF. Among them, LSTM has the best robustness and accuracy.

**Figure 10:**
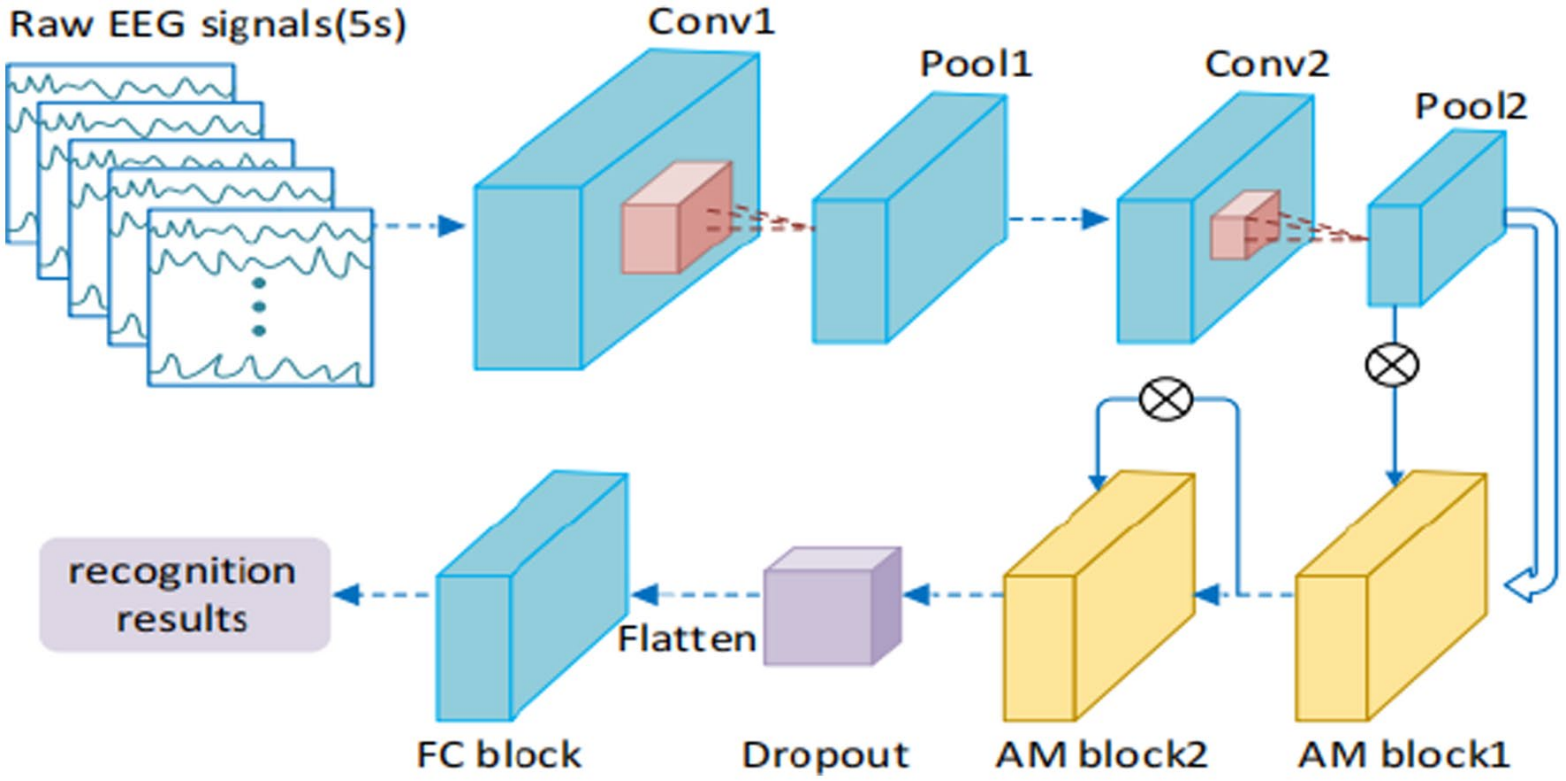
The flow of the 3DCANN algorithm (Liu et al.,2021). Reproduced with permission from IEEE. Licence Number: 5493600805113.

EEG signals are essentially multichannel time series signals. Thus, a more effective method for emotional recognition of EEG signals is to obtain the long-term dependence of EEG signals based on RNN. Li et al. (2020) proposed a BILSTM network framework based on multimodal attention, which is used to learn the best time characteristics, and inputted the learned depth characteristics into the DNN to predict the emotional output probability of each channel. A decision-level fusion strategy is used to predict the final emotion. The experimental results on AMIGOS dataset show that this method is superior to other advanced methods. Liu et al. (2017) proposed an emotion recognition algorithm model ground on multi-layer long short-term memory recurrent neural network (LSTM-RNN), which combines temporal attention (TA) and band attention (BA). Experiments on Mahnob-HCI database demonstrate the proposed method achieves higher accuracy and boosts the computational efficiency. Liu et al.(2021) studied an original algorithm named three-dimension convolution attention neural network (3DCANN) for EEG emotion recognition, which is composed of spatio-temporal feature extraction module and EEG channel attention weight learning module. Figure 10 presents the details of the used 3DCANN scheme. Alhagry et al. (2017) proposed an end-to-end deep learning neural network to identify emotions from original EEG signals. It uses LSTM-RNN to learn features from EEG signals and uses full connection layer for classification. Li et al. (2022) proposed a C-RNN model using CNN and RNN, and used multichannel EEG signals to identify emotions. Although the method based on RNN has great advantages in processing time series data and has made great achievements, it still has shortcomings in the face of multichannel EEG data. GRU or LSTM can connect the relationship between different channels through multichannel fusion, but this processing ignores the spatial distribution of EEG channels and cannot reflect the dynamics of the relationship between different channels.

**Figure 11:**
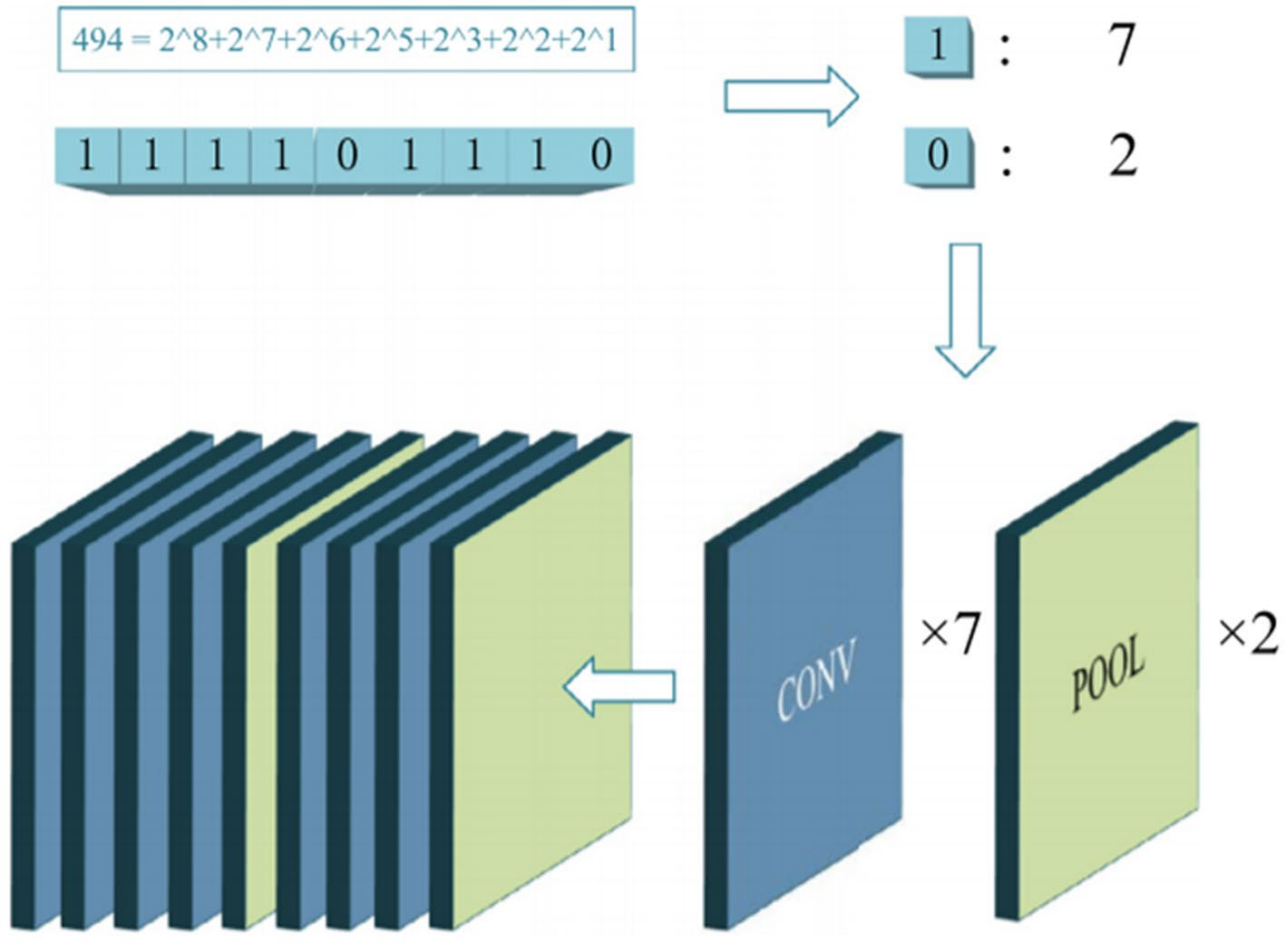
The schematic diagram of the GPSO algorithm (Gao et al., 2020). Reproduced with permission from Elsevier. Licence Number: 5493610089714.

Among various network algorithms, CNNs have a good ability to extract features of convolution kernels. They can extract information features by transferring each part of the image with multiple kernels. They have been widely used in image processing tasks. For EEG signal, they can process raw EEG data well and can be used for spectrum diagram. Considering that the use of CNN to train EEG data can reduce the effect of noise, most studies use CNN for the emotional recognition of EEG signals to reduce the complexity of training. Thodoroff et al. (2016) combined CNN and RNN to train robust features for automatic detection of seizures. Shamwell et al. (2016) explored a new CNN architecture with 4 convolutional layers and 3 fully connected layers to classify EEG signals. To reduce the over fitting of the model, Manor and Geva (2015) proposed a CNN model based on spatiotemporal regularization, which is used to classify single track EEG in RSVP (fast serial visual rendering). Sakhavi et al. (2015) proposed a parallel convolutional linear network, which is an architecture that can represent EEG data as dynamic energy input, and used CNN for image classification. Ren and Wu (2014) applied convolutional DBN to classify EEG signals. Hajinoroozi et al. (2017) used covariance learning to train EEG data for driver fatigue prediction. Jiao et al. (2018) proposed an improved CNN method for mental workload classification tasks. Gao et al. (2020) proposed a gradient particle swarm optimization (GPSO) model to achieve the automatic optimization of the CNN model. The experimental results show that the proposed method based on the GPSO-optimized CNN model achieve a prominent classification accuracy. Figure 11 presents the details of the used GPSO scheme.

CNN can use EEG to identify many human diseases. Antoniades et al. (2016) used deep learning to automatically generate features of EEG data in time domain to diagnose epilepsy. Page et al. (2016) conducted end-to-end learning through the maximum pool convolution neural network (MPCNN) and proved that transfer learning can be used to teach the generalized characteristics of MPCNN raw EEG data. Acharya et al. (2018b) proposed a five-layer deep CNN for detecting normal, pre seizure, and seizure categories.

The summary of recent state-of-the-art methods related to EEG-based emotion recognition system using machine learning and deep learning approaches is given in Table 2.

**Table 2:** Summary of EEG emotion recognition papers using Deep learning methods from 2017 to 2022.

Year	References	Stimulus	Classification methods	Emotion	Acc.(%)
2017	Alhagry et al. (2017)	DEAP	LSTM-RNN	Valence, Arousal, and Liking	Arousal: 85.65, Valence: 85.45, Liking: 87.99
2017	Li et al. (2017)	DEAP	CNN + RNN	Arousal and Valence	Arousal: 72.06, Valence: 74.12
2017	Liu et al. (2017)	Mahnob-HCI	LSTM-RNN	Valence, Arousal, and F1-score	Arousal: 73.1, Valence: 74.5; F1-score: Arousal: 72.3, Valence: 73.0
2018	Zhang T. et al. (2018)	SEED and CK+	STRNN	Positive, Negative, and Neutral	SEED: Overall Accuracy: 89.5 CK+: Overall Accuracy: 95.4
2018	Jiao et al. (2018)	The Sternberg memory task	Deep CNN	Types of mental load	Fused CNNs 1: 91.32 Fused CNNs 2: 92.37
2018	Song et al. (2018)	SEED and DREAMER	DGCNN	Positive, neutral and negative; Arousal, Valence, and Dominance	SEED: 90.40, DREAMER: Arousal: 84.54 Valence: 86.23 Dominance: 85.02
2018	Salama et al. (2018)	DEAP	3D-CNN	Arousal and Valence	Arousal: 88.49 Valence: 87.44
2019	Chao et al. (2019)	DEAP	CapsNet	Valence, Arousal and Dominance	Valence: 66.73 Arousal: 68.28 Dominance: 67.25
2019	Gonzalez et al. (2019)	DEAP, IAPS and DREAMER	CNN	Valence and Arousal	Single subject: Valence: 70.26 Arousal: 72.42
2019	Garg et al. (2019)	DEAP	Merged LSTM	Arousal, Valence, Liking and Dominance	Arousal: 83.85, Valence: 84.89 Liking: 80.72 Dominance: 84.37
2019	Wang et al. (2019)	SEED	DNNs	Positive, Negative, and Neutral	Overall Accuracy: 93.28
2019	Chen et al. (2019b)	DEAP	Bagging Tree (BT), SVM, LDA, Bayesian LDA, Deep CNN	Valence and Arousal	Valence: 99.97 (using CVCNN), Arousal: 99.58 (using GSLTCNN)
2019	Ma et al. (2019)	DEAP	multimodal residual LSTM	Arousal and Valence	Valence: 92.30 Arousal: 92.87
2019	Pandey and Seeja (2019)	DEAP	DNN	Arousal and Valence	Arousal: 61.25 Valance: 62.50
2020	Nakisa et al. (2020)	Audio-video Clips	ConvNet long short-term memory (LSTM; early and late fusion)	Low Arousal Positive, High Arousal Positive, Low Arousal Negative, High Arousal Negative	Overall accuracy: Early fusion:71.61 Late fusion: 70.17
2020	Nath et al. (2020)	DEAP	LSTM	Arousal and Valence	Valence: 94.69 Arousal: 93.13
2020	Gao et al. (2020)	Film clips	GPSO-optimized CNN	Fear, happiness, and sadness	Overall accuracy: 92.44 ± 3.60
2020	Joshi and Ghongade (2020)	Own dataset	BiLSTM	Positive, neutral and negative	Overall Accuracy:72.83
2020	Wei et al. (2020)	SEED	SRU	Positive, neutral and negative	Overall Accuracy:80.02
2020	Sharma et al. (2020)	DEAP and SEED	LSTM	Arousal and Valence Positive, neutral and negative	DEAP: 4 classes: 82.01 Arousal: 85.21 Valance: 84.16 SEED: 90.81
2020	Alhalaseh and Alasasfeh (2020)	DEAP	CNN, k-NN, NB, DT	Valence and Arousal	Overall accuracy: 95.20 (using CNN)
2020	Cui et al. (2020)	DEAP and DREAMR	Regional- Asymmetric Convolutional Neural Network (RACNN)	Valence and Arousal	Overall accuracy: 96.65 (Valence), 97.11 (Arousal)
2020	Hassouneh et al. (2020)	Own dataset	LSTM	Happy, fear, anger, sad, Surprise and disgust	Overall accuracy: 7.25
2020	Liu et al. (2020)	SEED	DECNN	Positive and negative	Overall accuracy: 97.56
2020	Li et al. (2020)	AMIGOS	Bidirectional LSTM-RNNs	Valence and Arousal	Arousal: F1-Score: 61.3, ACC: 73.5; Valence: F1-Score: 58.3, ACC: 67.8
2021	Topic and Russo (2021)	DEAP,DREAMER, SEED and AMIGOS.	CNN + SVM	Arousal and Valence; Positive and negative	DEAP: Arousal:77.7 andValence: 76.6 DREAMER: Arousal: 90.4 andValence: 88.2 AMIGOS: Arousal: 90.5 andValence: 78.4 SEED: 88.5
2021	Liu and Fu (2021)	DEAP	multi-channel feature fusion	SROCC and PLCC	SROCC: 78.9, PLCC: 84.3
2021	Sakalle et al. (2021)	Own dataset, DEAP and SEED	LSTM	Disgust, sadness, surprise, and anger Positive, negative, and neutral	DEAP: 91.38 SEED: 89.34 Own dataset: 4 class: 94.12 3 class: 92.66
2021	Huang et al. (2021)	DEAP	BiDCNN	Arousal and Valence	Subject-dependent Arousal:94.72 Valence: 94.38 Subject-independent Arousal: 63.94 Valence: 68.14
2022	Chowdary et al. (2022)	Own dataset	RNN, LSTM, and GRU	positive, negative, and neutral	average accuracy: RNN: 95, LSTM: 97, GRU:96
2022	Algarni et al. (2022)	DEAP	Bi-LSTM	arousal, valence and liking	average accuracy: valence: 99.45, arousal: 96.87, liking: 99.68
2022	Tuncer et al. (2022)	DREAMER	LEDPatNet19	arousal, dominance, and valance	valence: 94.58, arousal: 92.86, arousal: 94.44
2022	Li et al. (2022)	DEAP and SEED	ensemble learning	arousal and valence	DEAP average accuracy: Arousal: 65.70, valence: 64.22 SEED average accuracy: 84.44
2022	He et al. (2022)	DREAMER and DEAP	adversarial discriminative-temporal convolutional networks (AD-TCNs)	arousal and valence	DEAP average accuracy: Arousal: 64.33, valence: 63.25 DREAMER average accuracy: Arousal: 66.56, valence: 63.69
2022	Wang et al. (2022)	DEAP	2D CNN	arousal and valence	Average accuracy: Arousal: 99.99, valence: 99.98

## 5. Open challenges

To date, although a number of literature related to EEG emotion recognition using deep learning technology is reported, showing its certain advance, a few challenges still exist in this area. In the following, we discuss these challenges and opportunities, and point out potential research directions in the future.

### 5.1. Research on the basic theory of affective computing

At present, the theoretical basis of emotion recognition mainly includes discrete model and continuous model, as shown in Figure 3. Although they are related to each other, they have not formed a unified theoretical framework. The relationship between explicit information (such as happy, sad, and other emotional categories) and implicit information (such as the signal characteristics of different frequency bands of EEG signals corresponding to happy, sad, and other emotional categories) in emotional computing is worthy of further study. Digging out the relationship between them is extremely important for understanding the different emotional states represented by EEG signals.

### 5.2. EEG emotion recognition data sets

For EEG-based emotion recognition, most publicly available datasets for affective computing use images, videos, audio, and other external methods to induce emotional changes. These emotional changes are passive, which are different from the emotional changes that individuals actively produce in real scenes and may lead to differences in their EEG signals. Therefore, how to solve the difference between the external-induced emotional change and the internal active emotional change is a subject worthy of study.

Different individuals may not induce the same emotion for the same emotion-inducing video due to the differences in the physiology and psychology between different subjects. Although the same emotion is generated, the EEG signals will have some differences due to the physiological differences between individuals. To effectively solve the problem of individual differences, we can build a personalized emotional computing model from the perspective of individuals. However, building an emotion recognition model with better generalization ability is a relatively more economical solution because the collection and annotation of physiological signals will bring about a large cost. An effective method to improve the generalization ability of affective computing models is transfer learning (Pan et al., 2011). Therefore, how to combine the agent independent classifier model with the transfer learning technology may be a point worth being considered in the future.

The privacy protection of users’ personal information is an important ethical and moral issue in the Internet era. The EEG and other physiological signals collected in emotional computing belong to users’ private information, so privacy protection should be paid attention. At present, research in this area has only started (Cock et al., 2017; Agarwal et al., 2019).

### 5.3. EEG signal preprocessing and feature extraction

In the EEG signal acquisition experiment, many equipment are needed, and the noise acquisition should be minimized. However, EEG signal acquisition is more complex, and the acquisition results are often vulnerable to external factors. Therefore, collecting EEG signals with high efficiency and quality is an important part of affective computing. Effective preprocessing can remove the noise in the original EEG signal, improve the signal quality, and help feature extraction, which is another important part for affective computing.

The common features of EEG signal include power spectral density, differential entropy, asymmetric difference of differential entropy, asymmetric quotient of differential entropy, discrete wavelet analysis, empirical mode decomposition, empirical mode decomposition sample entropy (EMD_SampEn), and statistical features (mean, variance, etc.). How to extract appropriate features or fuse different features will have an important effect on affective computing models.

## 6. Conclusion

Multiple recent studies using deep learning have been conducted for EEG emotion recognition associated with promising performance due to the strong feature learning and classing ability of deep learning. This paper attempts to provide a comprehensive survey of existing EEG emotion recognition methods. The common open data sets of EEG-based affective computing are introduced. The deep learning techniques are summarized with specific focus on the common methods of emotional calculation of EEG signals, related algorithms. The challenges faced by emotional computing based on EEG signals and the problems to be solved in the future are analyzed and summarized.

## Author contributions

XW and YR contributed to the writing and drafted this article. ZL and JH contributed to the collection and analysis of existing literature. YH contributed to the conception and design of this work and revised this article. All authors contributed to the article and approved the submitted version.

## Funding

This work was supported by Hunan Provincial Natural Science Foundation of China (under Grant Nos. 2022JJ50148 and 2021JJ50082), Hunan Provincial Education Department Science Research Fund of China (under Grant Nos. 22A0625 and 21B0800), and National Innovation and Entrepreneurship Training for University of PRC (under Grant Nos. 202211528036 and 202211528023).

## Conflict of interest

The authors declare that the research was conducted in the absence of any commercial or financial relationships that could be construed as a potential conflict of interest.

## Publisher’s note

All claims expressed in this article are solely those of the authors and do not necessarily represent those of their affiliated organizations, or those of the publisher, the editors and the reviewers. Any product that may be evaluated in this article, or claim that may be made by its manufacturer, is not guaranteed or endorsed by the publisher.
